# Spin Laser Local Oscillators for Homodyne Detection in Coherent Optical Communications

**DOI:** 10.3390/mi12050573

**Published:** 2021-05-18

**Authors:** Nobuhide Yokota, Hiroshi Yasaka

**Affiliations:** Research Institute of Electrical Communication, Tohoku University, Sendai 980-8577, Japan; yasaka@riec.tohoku.ac.jp

**Keywords:** vertical-cavity surface-emitting laser, spin polarization, injection locking, local oscillator, coherent optical communication

## Abstract

We numerically investigate spin-controlled vertical-cavity surface-emitting lasers (spin-VCSELs) for local oscillators, which are based on an injection locking technique used in coherent optical communications. Under the spin polarization modulation of an injection-locked spin-VCSEL, frequency-shifted and phase-correlated optical sidebands are generated with an orthogonal polarization against the injection light, and one of the sidebands is resonantly enhanced due to the linear birefringence in the spin-VCSEL. We determine that the peak strength and peak frequency in the spin polarization modulation sensitivity of the injection-locked spin-VCSEL depend on detuning frequency and injection ratio conditions. As a proof of concept, 25-Gbaud and 16-ary quadrature amplitude modulation optical data signals and a pilot tone are generated, and the pilot tone is used for the injection locking of a spin-VCSEL. An orthogonally-polarized modulation sideband generated from the injection-locked spin-VCSEL is used as a frequency-shifted local oscillator (LO). We verify that the frequency-shifted LO can be used for the homodyne detection of optical data signals with no degradation. Our findings suggest a novel application of spin-VCSELs for coherent optical communications.

## 1. Introduction

Coherent optical communication systems have been gaining attention due to their benefits in a coherent detection scheme in combination with high-speed digital signal processing (DSP) for improving signal detection accuracy, frequency utilization efficiency, and tolerance to signal distortion [1,2]. In these systems, optical data signals are received in a coherent manner using a local oscillator (LO), and a coherent receiver in which in-phase and quadrature-phase components of the optical data signals can be detected. Thus, advanced modulation formats such as quadrature amplitude modulation (QAM), which have a high frequency utilization efficiency compared with the conventional on-off keying, can be used. A synchronization between the LO and the optical data signals can be obtained by using phase estimation techniques based on DSP at the receiver side [3,4,5]. However, large computations are required of the DSP when the modulation format is complex due to multi-level patterns, so hardware-based synchronization schemes are ideal in this case. Optical phase-locked loop (OPLL) circuits have been widely investigated for obtaining the synchronization in coherent optical communications [6,7]. However, conventional OPLL circuits are generally complex, and obtaining a wide bandwidth for the feedback loop is difficult. Additionally, both the OPLL and the DSP-based phase estimation schemes require narrow linewidth lasers for reducing the phase noise of the optical data signals, which leads to an increase in system cost.

We consider an injection locking scheme in which a semiconductor laser operating as an LO is injection-locked to a pilot tone transmitted with optical data signals. Synchronization can be carried out easily as this scheme does not require a narrow linewidth laser on the receiver side [8,9,10]. Liu et al. reported on the injection locking of a semiconductor laser to a residual optical carrier of orthogonal frequency division multiplexing signals and homodyne detection of the signals [9]. Although the use of the residual optical carrier for the injection locking is straightforward for the homodyne detection, a guard band in the optical data signals and detuned operation of a dual-parallel Mach–Zehnder modulator (DP-MZM), which leads to distortion of the optical data signals, are necessary. To circumvent these drawbacks, a combination of the injection-locked LO and a frequency shifter have been reported [10]. Hereafter, we refer to such an LO as a frequency-shifted LO.

**Figure 1:**
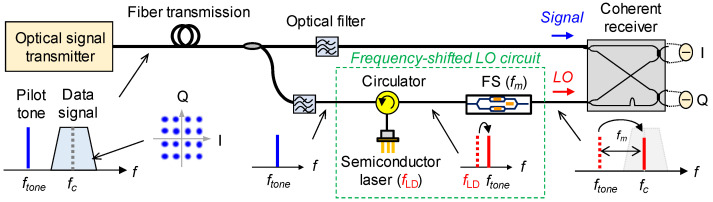
Schematic of coherent optical communication system based on frequency-shifted LO.

A schematic of the coherent optical communication system based on the frequency-shifted LO is shown in Figure 1. A pilot tone is added to data signals on the transmitter side and used for the injection locking of a semiconductor laser on the receiver side. The injection-locked semiconductor laser can be used as an LO for heterodyne detection with a frequency separation of |*f_tone_* − *f_c_*|, where *f_tone_* and *f_c_* are pilot tone frequency and center frequency of the data signals, respectively. However, heterodyne detection requires wide-band electrical components, which is unsuitable for high-baud-rate communications. As shown in Figure 1, homodyne detection is feasible when the frequency of the injection-locked semiconductor laser is shifted by *f_m_* using a frequency shifter (FS) to compensate for the frequency separation of |*f_tone_* − *f_c_*|. This method is applicable to the homodyne detection in the coherent optical communications and to carrier frequency conversion in beyond-5G wireless communications [11]. However, the configuration of a frequency-shifted LO, which requires a costly and complex FS, can be further simplified.

An approach to simplifying the frequency-shifted LO is to use the modulation sideband of directly modulated lasers (DMLs) to shift the LO frequency without using the FS. In particular, the photon-photon resonance effect, which occurs in external cavity structures and increases modulation sensitivity at high frequencies [12,13,14,15], may be useful for generating modulation sidebands with a high frequency separation. However, one of the modulation sidebands needs to be extracted by using a narrow-band optical filter, which is unsuitable for simplifying the configuration.

The approach we propose in this study is the use of spin-controlled vertical-cavity surface-emitting lasers (spin-VCSELs) [16,17,18,19,20,21,22,23]. The up-spin and down-spin electron densities in an active region can be freely modulated when ferromagnetic electron spin injectors for each spin-polarized electron are fabricated [24,25]. This results in the unique spin polarization modulation in which the difference between up-spin and down-spin electron densities is modulated while the total electron density kept constant. The spin polarization modulation leads to an increase in modulation sensitivity at high frequencies depending on linear birefringence in a spin-VCSEL [26,27,28] and orthogonally-polarized modulation sidebands [29]. Thus, a lasing frequency of the spin-VCSEL injection-locked to the pilot tone may be directly shifted with high efficiency and separated from the generated sidebands by using a simple polarization beam splitter.

In this study, we numerically investigate an application of spin-VCSELs to the frequency-shifted LO for coherent optical communications. As a proof of concept, we verify that an orthogonally-polarized modulation sideband generated by the spin polarization modulation of the injection-locked spin-VCSEL can be used as the frequency-shifted LO for the pilot-tone-assisted homodyne detection of 25-Gbaud 16-QAM signals.

## 2. Methods

### 2.1. Proposed Concept

**Figure 2:**
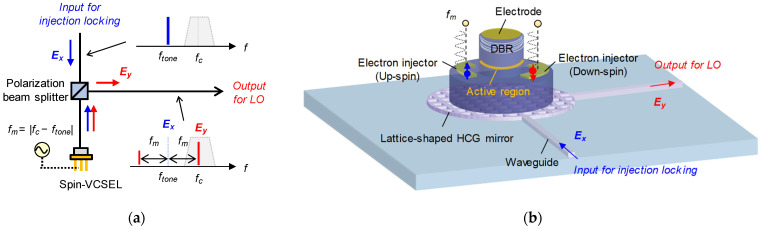
(**a**) Schematic of frequency-shifted LO based on injection-locked spin-VCSEL; (**b**) Example device structure of frequency-shifted LO with integrated configuration.

Figure 2a shows the proposed concept of frequency-shifted LOs based on injection-locked spin-VCSELs. The spin polarization of injected electrons into a spin-VCSEL is directly modulated by a sinusoidal signal under the injection locking to an input light with a linear polarization (*x* polarization in this case). Since modulation sidebands generated by the spin polarization modulation have orthogonal polarization against the optical carrier (DC lasing light component), the sidebands can be easily separated from the optical carrier by using a polarization beam splitter without a narrow-band optical filter. Additionally, one of the sidebands can be selectively enhanced by controlling the linear birefringence of the spin-VCSEL [29]. Therefore, we use the stronger sideband as a frequency-shifted LO for the homodyne detection of optical data signals. An example device structure of the frequency-shifted LO with an integrated configuration is illustrated in Figure 2b. The spin-VCSEL is integrated on a silicon photonics platform, and bottom of the VCSEL’s mirror consists of a lattice-shaped high-index contrast grating (HCG) instead of a conventional distributed Bragg reflector (DBR). The HCG can have a variety of characteristics such as a high-reflectivity and wide-band mirror, depending on its structure [30]. The lattice-shaped HCG can switch input/output port waveguides in accordance with the polarization of vertically injected light [31], so the polarization beam splitter in Figure 2a may be integrated with the HCG. Although obtaining a high polarization extinction ratio for each output port is challenging, the hybrid integration of the spin-VCSEL will be ideal for practical use.

### 2.2. Simulation Model

The spin-flip model [32] has been used for analyzing novel features of spin-VCSELs [33,34,35,36,37,38]. We used the following spin-flip rate equations, including an external light injection for simulating injection-locked spin-VCSELs and frequency-shifted LOs in coherent optical communication systems:

(1) $$\frac{dN^{\pm }}{dt}=\frac{I_{0}\pm I_{m}\sin2\pi f_{m}t}{eV}-v_{g}A_{g}\frac{N^{\pm }-N_{t}}{1+\varepsilon {\left|E^{\mp }\right|}^{2}}{\left|E^{\mp }\right|}^{2}-\frac{N^{\pm }}{\tau_{c}}\pm \frac{N^{-}-N^{+}}{\tau_{s}}$$

(2) $$\frac{dE^{\pm }}{dt}=\frac{1}{2}\left[\left(1+j\alpha \right)\left(\Gamma v_{g}A_{g}\frac{N^{\mp }-N_{t}}{1+\varepsilon {\left|E^{\pm }\right|}^{2}}-\frac{1}{\tau_{p}}\right)E^{\pm }+\left(\gamma_{a}-j2\pi \gamma_{p}\right)E^{\mp }\right]-j2\pi f_{d}E^{\pm }+\kappa E_{i}$$

where *E*^+^ and *E*^−^ are electric fields for right-handed (σ_+_) and left-handed (σ_−_) circular polarization modes, and *N*^+^ and *N*^−^ are up-spin and down-spin electron densities. Electric fields with *x* and *y* polarizations (*E_x_* and *E_y_*) are expressed as *E_x_* = (*E*^+^ + *E*^−^)/2 and *E_y_* = −*j*(*E*^+^ − *E*^−^)/2. *E_i_* is an electric field of the injection light with *x* polarization, and its value was set considering a parameter of injection ratio (IR) defined as IR = |*E_i_*|^2^/(|*E*^+^|^2^ + |*E*^−^|^2^). Definitions and values of other parameters are shown in Table 1. The parameter values were taken from 1.55-μm InAlGaAs VCSELs fabricated by RayCan Co., Ltd. (Suzhou, China) [39,40,41].

**Figure 3:**
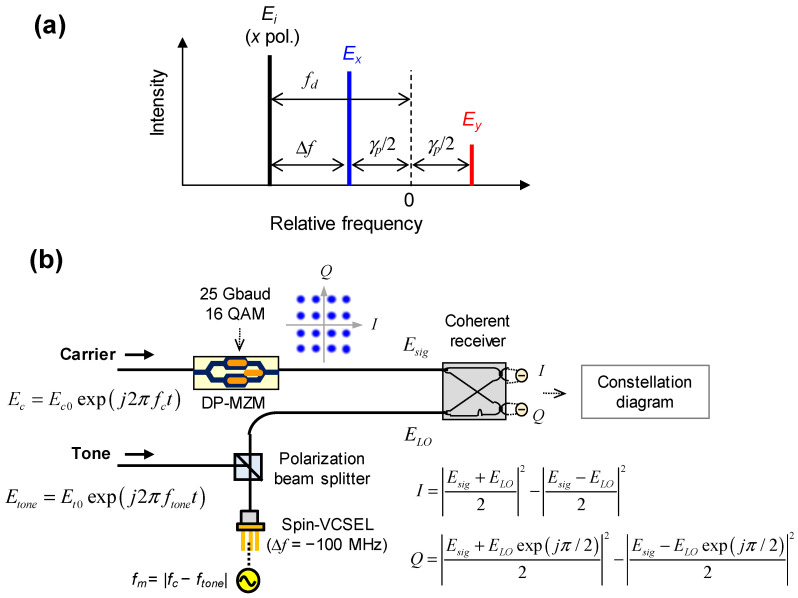
(**a**) Schematic of detuning condition for injection locking; (**b**) Schematic of proof-of-concept simulation of frequency-shifted LO based on injection-locked spin-VCSEL for homodyne detection.

The spin polarization modulation responses of the injection-locked spin-VCSEL and a proof-of-concept simulation were investigated using Equations (1) and (2). As shown in Figure 3a, we assume that the free-running spin-VCSEL is lasing with *x* polarization when injected electrons are not spin-polarized, and a detuning frequency of *E_i_* relative to *E_x_* is defined as ∆*f*. Under several injection locking conditions, the spin polarization of the spin-VCSEL was modulated by sinusoidal signals with frequencies of *f_m_*, and *I_m_* of 0.1 × *I*th was used for analyzing small signal responses. Then, we evaluated the corresponding modulation responses of degree of circular polarization (|*E*^+^|^2^ − |*E*^−^|^2^)/(|*E*^+^|^2^ + |*E*^−^|^2^). The concept of frequency-shifted LOs based on injection-locked spin-VCSELs was verified using the configuration shown in Figure 3b. Optical data signals with a 25-Gbaud 16-QAM pattern were generated by modulating an optical carrier (*E_c_*) with a DP-MZM model in [42]. A pilot tone (*E_tone_*) was used for injection locking of the spin-VCSEL with ∆*f* of −100 MHz. This slight detuning was to verify evident injection locking, i.e., a tight synchronization between the optical data signals and the frequency-shifted LO. Both *f_m_* and the frequency difference between the optical carrier and the pilot tone (*f_c_* − *f_tone_*) were set to 50 GHz, and *I_m_* of 1 × *I*th was used for strong sideband generation. The optical data signals and modulation sidebands extracted from a polarization beam splitter (i.e., *E_y_* component) are referred to as *E_sig_* and *E_LO_*, respectively. A coherent receiver with a 90° hybrid was used for the homodyne detection which extracts in-phase (*I*) and quadrature-phase (*Q*) components of the optical data signals. Finally, *I* and *Q* signals were evaluated in a constellation diagram and its error vector magnitude (EVM). Note that 16-QAM symbols in the constellation diagram will rotate if injection locking is not achieved due to the 100-MHz detuning. An ideal LO with frequency equal to *f_c_* without injection locking was also tested for comparison with the frequency-shifted LO based on the injection-locked spin-VCSEL.

## 3. Results

### 3.1. Modulation Response of Injection-Locked Spin-VCSEL

**Figure 4:**
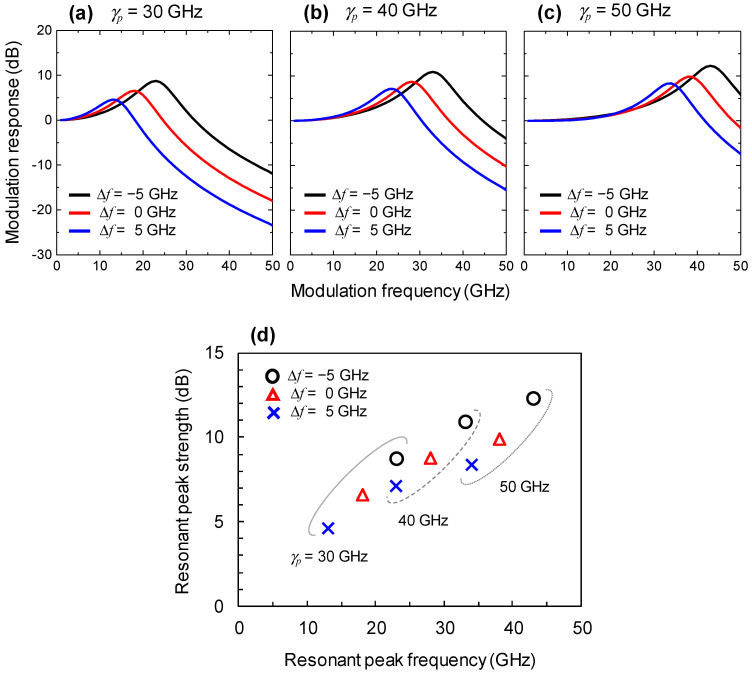
Spin polarization modulation responses of injection-locked spin-VCSELs calculated with IR of 10 dB. (**a**) *γ_p_* = 30 GHz; (**b**) *γ_p_* = 40 GHz; (**c**) *γ_p_* = 50 GHz. Black, red, and blue curves indicate results for ∆*f* of −5, 0, and 5 GHz, respectively. (**d**) Resonant peak strengths in relation to resonant peak frequencies observed in spin polarization modulation responses. Values for ∆*f* of −5, 0, and 5 GHz are indicated by the circles, triangles, and crosses, respectively. Values for *γ_p_* of 30, 40, and 50 GHz are grouped by solid, dashed, and dotted curves, respectively.

The modulation responses of an injection-locked spin-VCSEL with varying *γ_p_* and ∆*f* are shown in Figure 4. An IR of 10 dB was used for these simulations. As shown in Figure 4a, we verified that the ∆*f* value affects the resonant peak frequency and resonant peak strength of the spin polarization modulation response with *γ_p_* of 30 GHz. This tendency was also found with *γ_p_* of 40 and 50 GHz as shown in Figure 4b,c. Figure 4d shows a summary of the resonant peak frequency and strength of the spin polarization modulation responses. The higher *γ_p_* values contributed to increases in both the resonant peak frequency and strength of the modulation response. Thus, the strong birefringence in spin-VCSELs is useful for efficiently generating the orthogonally-polarized modulation sideband. The strong birefringence will be particularly desirable when data signals contain a wide bandwidth and the resulting frequency difference between the optical data signal and pilot tone (*f_c_* − *f_tone_*) requires a high frequency. The ∆*f* value also affected both the resonant peak frequency and the strength of the modulation response. ∆*f* nearly equal to zero will be the most practical and ideal situation since the locking range reaches the order of sub-gigahertz under low IRs [43]. Although high IRs are suitable for widening the locking range of the spin-VCSEL, direct use of a weak pilot tone without any optical amplifications is desirable for simplicity.

**Figure 5:**
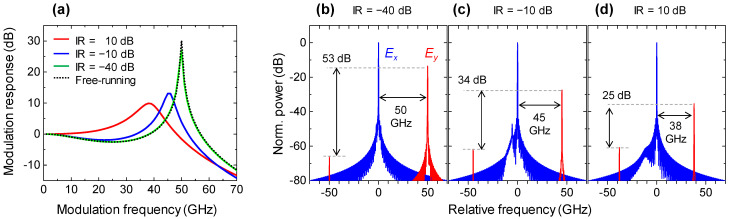
(**a**) Spin polarization modulation response under injection locking condition with different IRs and under free-running condition; Polarization-resolved optical spectra under spin polarization modulation of injection-locked spin-VCSEL. (**b**) IR = −40 dB, *f_m_* = 50 GHz; (**c**) IR = −10 dB, *f_m_* = 45 GHz; (**d**) IR = 10 dB, *f_m_* = 38 GHz. Blue and red curves indicate *x* and *y* polarization components, respectively. These are calculated with ∆*f* of 0 and *γ_p_* of 50 GHz.

The spin polarization modulation responses of the injection-locked spin-VCSEL with different IRs are shown in Figure 5a. ∆*f* = 0 and *γ_p_* = 50 GHz were used for these simulations. A low IR of −10 dB increased both the resonant peak frequency and the strength of the modulation response compared with when a high IR of 10 dB was used. Moreover, the response shape with a fairly low IR of −40 dB almost overlapped with that under the free-running condition. This tendency is reasonable because the free-running condition is a lower limit of the IR, and the resonant peak frequency is dominated by the *γ_p_* value. Polarization-resolved optical spectra of the injection-locked spin-VCSEL with the IR of −40, −10, and 10 dB under spin polarization modulation are shown in Figure 5b–d. The *f_m_* of 50, 45, and 38 GHz are used for Figure 5b–d, respectively. The modulation sidebands have *y* polarization in contrast to the lasing optical carrier of *x* polarization. As shown in Figure 5b, the sideband intensity at 50 GHz is 53 dB stronger than that at −50 GHz, which is attributed to a relative frequency difference between the lasing polarization mode and the non-lasing residual polarization mode in a birefringent cavity. This asymmetric characteristic can imitate a single sideband modulation ideal for frequency shifting without using a narrow-band optical filter. Note that the modulation sideband includes effects of intensity modulations and chirps in the spin-VCSEL [44], which means that the sideband intensity depends on *α* value. The ratio between the stronger and weaker sideband intensities decreases when the IR increases as shown in Figure 5c,d. These results show that efficient generation of orthogonally-polarized single modulation sideband is feasible due to the spin polarization modulation of the injection-locked spin-VCSEL under a low IR.

### 3.2. Proof of Concept

**Figure 6:**
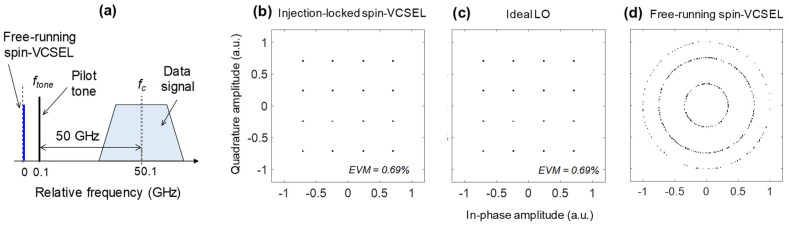
(**a**) Schematic relation among *f_c_*, *f_tone_*, and data signal. Constellation diagrams of detected 25-Gbaud 16-QAM signals. (**b**) With injection-locked spin-VCSEL; (**c**) With ideal LO; (**d**) With free-running spin-VCSEL.

Finally, a proof-of-concept simulation was conducted using the configuration shown in Figure 3b. We used the following variable parameters: *I_m_* = 1 × *I*th, *f_m_* = 50.0 GHz, *γ_p_* = 50.0 GHz, *f_d_* = 24.9 GHz, ∆*f* = −100 MHz, *f_c_* = 50.1 GHz, and *f_tone_* = 100 MHz. The relation among *f_c_*, *f_tone_*, and data signal is schematically shown in Figure 6a. A constellation diagram of 25-Gbaud 16-QAM signals detected with the spin-VCSEL operating as a frequency-shifted LO is shown in Figure 6b. The 16-QAM signal pattern was clearly observed with a low enough EVM of 0.69%. This EVM value is dominated by nonlinear signal distortion in the DP-MZM used for the optical data signal generation. The same EVM value was also observed when the ideal LO was used (Figure 6c). These results indicate that the injection-locked spin-VCSEL can be used for the frequency-shifted LO in principle. As shown in Figure 6d, the constellation rotated when the pilot tone for the injection locking of the spin-VCSEL was removed due to the 100-MHz frequency difference between them. This result verifies that tight synchronization was obtained by the injection locking.

## 4. Discussion and Prospects

Although the proposed scheme was determined to be useful for obtaining LO for homodyne detection, the role of amplified spontaneous emission (ASE) noise of Er-doped fiber amplifiers which are added into optical signals should be further investigated for the proposed scheme. The ASE noise usually degrades the EVM of the detected signal when the injection-locked semiconductor laser is used as an LO [8]; this effect should be taken into account for practical application. The effect of unnecessary current modulation during the spin polarization modulation on the homodyne detected signal should also be clarified.

It is worth noting that several technical advancements are required for the proposed device function. First, the spin-VCSELs need to be integrated with silicon photonics-based components such as low-loss waveguides. Although conventional VCSELs can be fabricated on silicon [45,46,47], fabricating spin-VCSELs on silicon requires additional technologies such as electrical spin injections and transports [48,49,50,51,52,53,54]. Electrical spin injection into long wavelength active regions such as InGaAsP and InAlGaAs quantum wells is required, since the bandgap of silicon is 1.1 eV and well-studied GaAs quantum wells are not compatible. The up-spin and down-spin electron injectors may be obtained by manipulating remanence of vertically-magnetized ferromagnetic metal contacts such as Fe/Tb multilayer/Schottky [55] and FePt/MgO contacts [56] using a magnetic head similar to the case of hard disk drives. The use of spin precession of drifting electrons due to spin–orbit interactions [57] may be another approach when transversely-magnetized ferromagnetic metal contacts [25] are used in combination. Second, a polarization selective function of the device, for example, polarization extinction ratio of the lattice-shaped HCG is important for practical use. When the optical carrier remains after the polarization selective component, it causes cross talk at the stage of polarization hybrid coherent detection. Third, birefringence controls of spin-VCSELs including theoretical modeling are important for efficient generation of orthogonally-polarized modulation sideband with a high frequency [58,59,60,61,62]. Since the sensitivity of spin polarization modulation is subject to birefringence, efficient generation of an orthogonally-polarized modulation sideband requires birefringence control with accuracy in the order of a few gigahertz. Birefringence controls of spin-VCSELs on silicon will be particularly challenging since heterogeneous integration of III-V materials on silicon is usually conducted by wafer bonding techniques [47] which tend to induce non-uniform stress distribution. Electrical birefringence tuning with sub-gigahertz accuracy [63,64] is one such promising approach. Since spin-VCSELs have a possibility of improving optical signal quality [65] and high-speed modulations [28], they are expected to be used also for optical data signal generators in the coherent optical communication systems. Ideas based on electrical spin injection into silicon [66] may provide other interesting degrees of freedom to the spin-VCSEL.

## 5. Conclusions

We numerically investigated injection-locked spin-VCSELs for a frequency-shifted LO in coherent optical communication systems. The spin polarization modulation responses of the injection-locked spin-VCSEL indicated a resonance feature, and its peak frequency and peak strength were controlled by the injection ratio and detuning frequency. The modulation sensitivity was maximized at the lower extrema of the injection ratio corresponding to the free-running condition. The proof-of-concept simulation verified that 25-Gbaud 16-QAM signals can be homodyne detected by using the injection-locked spin-VCSEL as the frequency-shifted LO.

## Figures and Tables

**Table 1:** Simulation parameters. *I*th denotes threshold current.

Symbol	Meaning	Value
*I*_0_	Static current	2 *I*th
*I*_m_	Current modulation coefficient	0.1 × *I*th or 1 × *I*th
*f_m_*	Modulation frequency	Variable
*V*	Cavity volume	2.5 × 10^−18^ m^3^
*v_g_*	Group velocity	9.3 × 10^7^ m/s
*A_g_*	Differential gain coefficient	1.2 × 10^−20^ m^2^
*N_t_*	Transparency carrier density	3.8 × 10^24^ m^−3^
*ε*	Gain compression factor	1.0 × 10^−24^ m^3^
*τ_c_*	Carrier lifetime	1.2 ns
*τ_s_*	Electron spin relaxation time	20 ps
*τ_p_*	Photon lifetime	19 ps
*α*	Linewidth enhancement factor	2.8
Γ	Confinement factor	0.05
*γ_a_*	Dichroism	0.5 GHz
*γ_p_*	Linear birefringence	Variable
*f_d_*	Detuning frequency	Variable
*κ*	Coupling rate	1.2 × 10^11^ s^−1^

## Data Availability

The data presented in this study are available on request from the corresponding author.
